# Improvement of marker-based predictability of Apparent Amylose Content in *japonica* rice through *GBSSI* allele mining

**DOI:** 10.1186/1939-8433-7-1

**Published:** 2014-01-02

**Authors:** Chiara Biselli, Daniela Cavalluzzo, Rosaria Perrini, Alberto Gianinetti, Paolo Bagnaresi, Simona Urso, Gabriele Orasen, Francesca Desiderio, Elisabetta Lupotto, Luigi Cattivelli, Giampiero Valè

**Affiliations:** 1 Rice Research Unit, CRA-Consiglio per la Ricerca e la Sperimentazione in Agricoltura, S.S. 11 to Torino, Km 2,5, 13100 Vercelli, Italy; 2 Genomics Research Centre, CRA-Consiglio per la Ricerca e la Sperimentazione in Agricoltura, Via S. Protaso 302, 29017 Fiorenzuola d’Arda, Piacenza, Italy; 3 Department of Plant Biology and Crop Production, CRA-Consiglio per la Ricerca e la Sperimentazione in Agricoltura, Roma, Italy

**Keywords:** Apparent amylose content, Grain shape characters, Marker-Assisted Selection (MAS), Molecular markers, Re-sequencing, Rice (*Oryza sativa* L.)

## Abstract

**Background:**

Apparent Amylose Content (AAC), regulated by the *Waxy* gene, represents the key determinant of rice cooking properties. In occidental countries high AAC rice represents the most requested market class but the availability of molecular markers allowing specific selection of high AAC varieties is limited.

**Results:**

In this study, the effectiveness of available molecular markers in predicting AAC was evaluated in a collection of 127 rice accessions (125 *japonica* ssp. and 2 *indica* ssp.) characterized by AAC values from glutinous to 26%. The analyses highlighted the presence of several different allelic patterns identifiable by a few molecular markers, and two of them, i.e., the SNPs at intron1 and exon 6, were able to explain a maximum of 79.5% of AAC variation. However, the available molecular markers haplotypes did not provide tools for predicting accessions with AAC higher than 24.5%. To identify additional polymorphisms, the re-sequencing of the *Waxy* gene and 1kbp of the putative upstream regulatory region was performed in 21 genotypes representing all the AAC classes identified. Several previously un-characterized SNPs were identified and four of them were used to develop dCAPS markers.

**Conclusions:**

The addition of the SNPs newly identified slightly increased the AAC explained variation and allowed the identification of a haplotype almost unequivocally associated to AAC higher than 24.5%. Haplotypes at the *waxy* locus were also associated to grain length and length/width (L/W) ratio. In particular, the SNP at the first intron, which identifies the *Wx*^*a*^ and *Wx*^*b*^ alleles, was associated with differences in the width of the grain, the L/W ratio and the length of the kernel, most likely as a result of human selection.

## Background

Rice (*Oryza sativa* L.) is the most important staple food for over half of the world population. Rice quality is primarily influenced by starch which is composed of two polysaccharides: amylose and amylopectin. The percentage of amylose on total starch, measured as Apparent Amylose Content (AAC), is the key determinant of rice cooking properties. High amylose varieties, like risotto varieties, cook dry with firm and separate grain; while low amylose cultivars (cvs.) are tender, glossy and cohesive after cooking. Suggested classification of amylose content identified classes as waxy (0–5%), very low (5–12%), low (12–20%), intermediate (20–25%), and high (25–33%), even considering that commercially rice is classified by amylose content as either low (less than 20% amylose), medium (21–25%) and high (26–33%) (Juliano 1992; Suwannaporn et al. 2007).

Over the past several decades, various methods have been reported for the determination of amylose content, including iodine binding, near infrared spectroscopy, size-exclusion chromatography and most recently, asymmetric field flow fractionation (Juliano 1971; Wesley et al. 2003; Ward et al. 2006; Chiaramonte et al. 2012). However, none of these methods is cost-effective in terms of high throughput screening (Caffagni et al. 2013) and the iodine binding represents the only one which has been validated for routine use (Fitzgerald et al. 2009). The utilization of marker-assisted selection (MAS) could overcome the shortcomings of amylose content measurements and, considering its lower cost and higher throughput, can be applied as a selection tool in the early phases of the breeding, while AAC direct analysis would require seeds setting. In addition, DNA markers for AAC also allow homozygous and heterozygous plants to be readily distinguished and often provides a more definitive way of classifying *Granule-Bound Starch Synthase* (*GBSS*) alleles compared to AAC assays since it avoids complications such as modifier genes, cytoplasmic factors, as well as environmental effects such as differences in temperature during grain development (McKenzie and Rutger 1983; Asaoka et al. 1985; Kumar and Khush 1987).

Amylose synthesis in developing seeds is primarily regulated by the *Waxy* (*Wx*) gene which encodes the Granule-Bound Starch Synthase I (GBSSI) enzyme and the level of grain amylose is directly associated to the amount of GBSSI in the endosperm (Mikami et al. 2008). The *Waxy* gene is located on chromosome 6 and consists of 13 exons and 12 introns. Two wild type alleles, *Wx*^*a*^, primarily found in *indica* subspecies, and *Wx*^*b*^, mainly found in *japonica* subspecies, have been found to predominate at the *waxy* locus for high and low AAC respectively (Dobo et al. 2010). The difference between the two alleles is related to the presence in *Wx*^*b*^ of a G to T Single Nucleotide Polymorphism (SNP) at the 5′ splice site of the first 1,124 bp long intron, localized 1,164 bp upstream the start codon (haplotype AG**T**T instead of AG**G**T). Most of the waxy and low AAC cvs. screened so far carry this polymorphism which results, for *Wx*^*b*^, in the reduction of pre-mRNA splicing efficiency and promotion of alternative splicing at cryptic sites in exon 1, leading to a decreased production of functional enzymes and causing the glutinous and low amylose phenotypes (Wang et al. 1995; Ayres et al. 1997; Bligh et al. 1998; Cai et al. 1998; Isshiki et al. 1998; Larking and Park 1999). As the SNP implies the generation of a restriction site, a Cleaved Amplified Polymorphic Sequence (CAPS) marker was developed and largely used to discriminate between high (G allele) and low (T allele) amylose varieties. Another diagnostic molecular marker widely utilized is the RM190 CT_n_ microsatellite, located in the 5′ Untranslated Region (UTR) of *Waxy* exon 1 (Bligh et al. 1995). Seven variants of this marker are known enabling the discrimination between low, intermediate and high AAC genotypes (Ayres et al. 1997; Shu et al. 1999; Bergman et al. 2001; Tan and Zhang 2001). However, failures in detecting specific correlation between the number of RM190 repeats and an AAC group were frequently observed, suggesting that this microsatellite is simply a closely linked marker rather than the causal mutation for AAC variation (Ayres et al. 1997; Dobo et al., 2010).

The ever-increasing demand of rice varieties with higher eating quality drives to the identification of superior and novel alleles to be used in breeding programs. The allele mining approach, based on the sequencing of different alleles of a single gene in different genotypes within a *specie*, has been largely applied to mine the sequence diversification at the level of the key genes for quality with the aim of studying the available diversity and/or developing allele-specific molecular markers for Marker-Assisted Selection (MAS). One example in rice is the identification of 24 SNPs and one InDel mutation obtained through the comparison of the *Starch Synthase II* (*SSII*) gene sequence in 30 rice genotypes. The polymorphisms were then utilized for marker-assisted backcross breeding programs (Bao et al. 2006). Moreover, Takanokai and co-workers (2009) compared the sequence of the *GS3* gene, responsible of grain size, in 54 rice cvs. and identified 86 SNPs and 20 InDels. The allele mining for the rice fragrant gene *badh2* also allowed the development of diagnostic molecular markers (Shi et al. 2008).

Allele mining experiments applied to the *Waxy* gene led to the identification of five different allelic variants. Mikami et al. (2008) studied the allelic diversification at the *Wx* locus in Asian rice associating different alleles to grain amylose content alterations. They identified the *Wx*^*op*^ allele in *indica* varieties from India, Nepal, Indonesia and China showing an opaque and chalky endosperm with a very low amylose content. This allele is characterized by an A to G SNP in exon 4 at position +715 from the ATG causing an Asp to Gly aminoacid change. The allele *Wx*^*in*^ was frequent in accessions belonging to an aromatic group and to tropical *japonica* which exhibited an intermediate AAC and is determined by a non-conservative mutation in exon 6 at position +1,083. The mutation alters a Tyr to Ser in the active site of the enzyme reducing its specific activity (Dobo et al. 2010). The last allele analyzed by Mikami and co-workers (2008), the *wx* allele, is present only in waxy varieties and is characterized by a 23 bp duplication in the second exon, 100 bp downstream the ATG, causing a premature stop codon which inactivates the *Waxy* gene. A minor allele, represented by *Wx*^*mq*^, was identified in the low AAC rice cv. Milky Queen and was characterized by two base changes within the coding region: G to A in exon 4 and T to C in exon 5. Each of these mutations generated missense aminoacid substitutions (Sato et al. 2002). One additional low AAC-associate allele, *Wx*^*hp*^, was identified by Liu et al. (2009) in a few low AAC Yunnan landraces. An A to G SNP occurring in exon 2 at position +497 causes an Asp to Gly substitution resulting in a reduction of the activity of GBSSI.

Following the discovery of this variability at the *Wx* locus, additional AAC class-specific molecular markers were developed. The combination of the *Wx*^*in*^ SNP in exon 6 and a SNP in exon 10, which consists of a C/T non-conservative SNP, with the RM190 microsatellite in exon 1 enabled the discrimination between intermediate and high AAC in a small number of genotypes (Larkin and Park 2003). More recently, Dobo and co-workers (2010) performed the same analysis increasing the number of genotypes and identified three allelic groups based on the combination of the three SNPs explaining 89.2% of the variation in AAC among 85 US varieties and 93.8% of the variation in 279 European accessions.

To date, the attention has been focalised in the identification of *Wx* genetic variants explaining amylose reduction in the endosperm of intermediate, low and waxy genotypes and the molecular markers used so far can discriminate only waxy, low, intermediate and high AAC. There are no markers explaining the different percentages of seed amylose within the high amylose class and further elucidations are needed to improve rice quality particularly in occidental countries where dry and firm rice is largely preferred.

In this study a collection of 127 rice accessions (125 *japonica* ssp. and 2 *indica* ssp.) was analyzed for AAC leading to the identification of the AAC classes low, intermediate and high. Effectiveness of available markers diagnostic for Apparent Amylose Content was verified and a low predictability within the high AAC class was observed. To discover new molecular markers associated to the different AACs, twenty-one genotypes representing each AAC class were selected and subjected to the re-sequencing of the *waxy* locus. New SNPs were identified in four high AAC accessions and used to develop new SNP-based molecular markers. Moreover, un-expected associations between grain shape characters and polymorphisms associated to the *waxy* locus were identified and analyzed.

## Results

### AAC assessment in a rice germplasm collection

**Figure 1:**
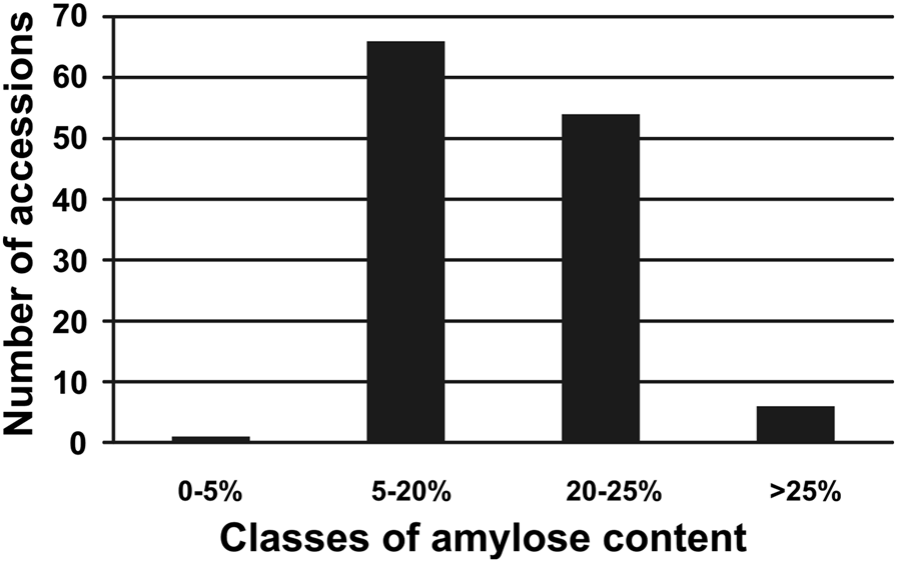
Frequency distribution of amylose content classes in the analyzed germplasm collection: Waxy (0-5%), low amylose (5-20%), intermediate (20-25%) and high amylose (>25%).

A collection of 125 temperate *japonica* and two *indica* rice accessions originated from different rice cultivation areas was evaluated for AAC and four classes were identified, ranging from waxy to high AAC (Table 1). No accessions of the very low AAC class (5–12%) were detected; high frequency was instead observed for low and intermediate amylose classes (Figure 1) while a low frequency of the high amylose class was highlighted. Accessions showing AAC higher than 20% (60 in total) originated from very different countries (20 from USA, 11 from Italy, 4 from Portugal, 3 from Spain, 2 from France and the remaining from other countries), indicating that the trait conferring a relatively high amylose content was selected in different rice cultivation areas.

**Table 1:** Germplasm collection of 127 rice accessions

**Accession**	**AAC (%)**	**EU Classification**	**Group**	**Origin**	**Maturity time (days)**	**Grain length (mm)**^**a**^	**Grain width (mm)**^**a**^	**Length to width ratio**	**RM190**	** T/G intron 1**	** A/C exon 6**	**_1514 G/T promoter**	**1801 T/C exon 9**	**2282 A/G intron 10**	**2806 C/T intron 12**
CALMOCHI 101	-	Medium	Jap	USA	158	5,26	2,97	1,77	17	T	A	G	T	A	C
PREVER	14,92	Long B	Jap	ITA	141	7,22	2,16	3,34	18	T	A	G	T	A	C
MEJANES 2	15,21	Long B	Jap	FRA	144	8,24	2,28	3,61	18	T	A	G	T	A	C
YRL 196	15,55	Medium	Jap	AUS	142	5,90	2,61	2,26	19	T	A	G	T	A	C
DELTA	15,60	Long A	Jap	FRA	139	8,16	2,90	2,81	18	T	A	G	T	A	C
LOMELLINO	15,77	Medium	Jap	ITA	129	5,67	3,22	1,76	18	T	A	G	T	A	C
SOURE	15,86	Long A	Jap	PRT	149	7,56	2,88	2,63	18	T	A	G	T	A	C
CAMPINO	15,90	Medium	Jap	PRT	145	6,19	3,39	1,83	18	T	A	G	T	A	C
EUROSIS	16,11	Long A	Jap	ITA	149	6,73	2,52	2,67	18	T	A	G	T	A	C
FAMILIA 181	16,27	Long A	Jap	PRT	159	7,05	2,85	2,47	18	T	A	G	T	A	C
TAICHUNG 65	16,29	Long A	Jap	TWN	149	6,58	2,66	2,47	18	T	A	G	T	A	C
SMERALDO	16,34	Long A	Jap	ITA	159	6,89	2,64	2,61	18	T	A	G	T	A	C
YRM 6-2	16,40	Medium	Jap	AUS	160	5,93	2,90	2,04	19	T	A	G	T	A	C
TIMICH 108	16,42	Round	Jap	ROM	127	4,92	3,13	1,57	17	T	A	G	T	A	C
GIOVANNI MARCHETTI	16,53	Medium	Jap	ITA	140	5,76	3,29	1,75	18	T	A	G	T	A	C
TOPAZIO	16,55	Medium	Jap	PRT	138	5,70	3,31	1,72	18	T	A	G	T	A	C
ZENA	16,57	Long B	Jap	ITA	144	7,49	2,16	3,47	18	T	A	G	T	A	C
RIBE 253	16,71	Long A	Jap	ITA	149	6,91	2,88	2,40	18	T	A	G	T	A	C
PIEMONTE	16,98	Long A	Jap	ITA	149	6,21	3,25	1,91	19	T	A	G	T	A	C
RPC 12	17,03	Round	Jap	CHN	141	5,01	3,11	1,61	18	T	A	G	T	A	C
RONCOLO	17,06	Medium	Jap	ITA	144	5,69	3,22	1,77	18	T	A	G	T	A	C
SLAVA	17,23	Medium	Jap	BGR	144	6,14	3,38	1,82	18	T	A	G	T	A	C
SELN 244 A6-20	17,29	Medium	Jap	AUS	154	5,34	3,17	1,68	18	T	A	G	T	A	C
BONNI	17,47	Long A	Jap	ITA	143	7,72	2,83	2,73	19	T	A	G	T	A	C
OSCAR	17,56	Long A	Jap	PRT	166	7,12	2,40	2,97	18	T	A	G	T	A	C
PERLA	17,67	Round	Jap	ITA	150	5,14	2,88	1,78	18	T	A	G	T	A	C
ROXANI	17,72	Long A	Jap	GRC	162	7,01	2,96	2,37	18	T	A	G	T	A	C
SELENIO	17,75	Round	Jap	ITA	155	5,01	2,90	1,73	17	T	A	G	T	A	C
ARBORIO	17,79	Long A	Jap	ITA	149	7,21	3,50	2,06	18	T	A	G	T	A	C
GRITNA	17,83	Long A	Jap	ITA	140	7,21	2,94	2,45	18	T	A	G	T	A	C
S 101	17,85	Medium	Jap	USA	153	5,28	2,93	1,80	18	T	A	G	T	A	C
AUGUSTO	18,06	Long A	Jap	ITA	143	6,86	2,69	2,55	18	T	A	G	T	A	C
ARIETE	18,08	Long A	Jap	ITA	145	6,81	2,64	2,58	18	T	A	G	T	A	C
GIZA 177	18,10	Medium	Jap	EGY	158	5,36	2,90	1,85	17	T	A	G	T	A	C
SAVIO	18,11	Long A	Jap	ITA	151	6,57	2,60	2,53	18	T	A	G	T	A	C
LOTO	18,20	Long A	Jap	ITA	139	6,51	2,95	2,21	18	T	A	G	T	A	C
KORAL	18,27	Long A	Jap	ITA	151	6,89	2,74	2,51	18	T	A	G	T	A	C
T 757	18,30	Long A	Jap	IND	162	6,22	3,20	1,94	18	T	A	G	T	A	C
SHSS 53	18,44	Long A	Jap	ESP	158	6,63	2,96	2,24	18	T	A	G	T	A	C
SCUDO	18,49	Long B	Jap	ITA	163	7,53	2,29	3,29	18	T	A	G	T	A	C
CHACARERO	18,55	Long A	Jap	USA	164	7,84	3,00	2,61	18	T	A	G	T	A	C
M 202	18,55	Medium	Jap	USA	152	6,00	2,82	2,13	18	T	A	G	T	A	C
MELAS	18,61	Long B	Jap	GRC	154	7,04	2,34	3,01	18	T	A	G	T	A	C
GUITA	18,62	Medium	Jap	PHL	162	5,90	2,98	1,98	18	T	A	G	T	A	C
IR56381-139-2-2	18,65	Long A	Jap	ARG	160	6,34	3,05	2,08	18	T	A	G	T	A	C
LA PLATA GUANEJAN	18,65	Long A	Jap	PHL	161	7,53	3,01	2,50	18	T	A	G	T	A	C
MARATELLI	18,66	Medium	Jap	ITA	145	5,61	3,13	1,79	18	T	A	G	T	A	C
KULON	18,76	Long A	Jap	RUS	147	6,55	2,91	2,25	18	T	A	G	T	A	C
ESTRELA IRRADIADO	18,80	Long A	Jap	PRT	154	6,50	2,93	2,22	18	T	A	G	T	A	C
BENGAL	18,81	Long A	Jap	USA	163	6,11	2,74	2,23	18	T	A	G	T	A	C
CENTAURO	18,86	Round	Jap	ITA	155	5,60	3,36	1,67	18	T	A	G	T	A	C
LUXOR	18,88	Long A	Jap	ITA	158	6,48	3,02	2,15	18	T	A	G	T	A	C
CIGALON	18,92	Medium	Jap	FRA	140	5,38	3,14	1,71	18	T	A	G	T	A	C
ERCOLE	19,00	Long A	Jap	ITA	157	7,02	2,88	2,44	19	T	A	G	T	A	C
THAIPERLA	19,09	Round	Jap	USA	161	5,55	3,25	1,71	18	T	A	G	T	A	C
S. ANDREA	19,14	Long A	Jap	ITA	157	6,92	3,29	2,10	18	T	A	G	T	A	C
SIS R215	19,17	Long A	Jap	ITA	153	7,30	2,57	2,84	18	T	A	G	T	A	C
COLINA	19,25	Round	Jap	ESP	161	5,18	3,06	1,69	18	T	A	G	T	A	C
FLIPPER	19,28	Medium	Jap	ITA	152	5,99	3,01	1,99	18	T	A	G	T	A	C
ITALPATNA 48	19,34	Long A	Jap	ITA	151	7,12	2,60	2,74	17	T	A	G	T	A	C
M 204	19,42	Long A	Jap	USA	156	6,19	2,85	2,17	18	T	A	G	T	A	C
SAKHA 102	19,43	Medium	Jap	EGY	166	5,59	2,90	1,93	17	T	A	G	T	A	C
CENTURY PATNA	19,52	Long A	Jap	USA	157	8,00	3,15	2,54	18	T	A	G	T	A	C
SALVO	19,67	Long B	Jap	ITA	154	7,36	2,12	3,47	18	T	A	G	T	A	C
PECOS	19,77	Medium	Jap	USA	161	5,62	2,81	2,00	18	T	A	G	T	A	C
89 AXHVA-6	19,97	Round	Jap	ARG	166	5,69	3,13	1,82	18	T	A	G	T	A	C
Upla 104	19,97	Long A	Jap	ARG	168	7,95	3,02	2,63	18	T	A	G	T	A	C
CT 23	20,03	Long B	Jap	COL	157	7,93	2,45	3,24	18	T	A	G	T	A	C
FIDJI	20,05	Long B	Jap	PHL	163	7,88	2,23	3,53	18	T	A	G	T	A	C
S 102-2	20,09	Long A	Jap	USA	164	5,62	3,23	1,74	18	T	A	G	T	A	C
MAIORAL	20,18	Long A	Jap	PRT	154	7,96	2,83	2,81	18	T	A	G	T	A	C
BELOZEM	20,21	Medium	Jap	BGR	157	6,04	3,25	1,86	18	T	A	G	T	A	C
SUWEON 280	20,34	Long A	Jap	KOR	-	6,00	2,64	2,72	18	T	A	G	T	A	C
GRAAL	20,40	Long B	Jap	FRA	149	7,63	2,23	3,42	18	T	A	G	T	A	C
CAPATAZ	20,53	Long A	Jap	ESP	157	6,31	3,23	1,95	18	T	A	G	T	A	C
UPLA 80	20,54	Long B	Jap	ARG	142	7,81	2,12	3,68	18	T	A	G	T	A	C
ROTUNDUS	20,65	Long A	Jap	HUN	139	8,75	3,46	2,53	17	G	C	T	T	A	C
ITALPATNA X MILYANG	20,96	Long A	Jap	PRT	150	6,69	2,72	2,46	18	T	A	G	T	A	C
UPLA 91	21,21	Long A	Jap	ARG	140	7,11	2,43	2,93	18	T	A	G	T	A	C
ANTONI	21,27	Long A	Jap	BGR	131	6,49	2,91	2,23	17	G	C	T	T	A	C
GOOLARAH	21,30	Long B	Jap	AUS	174	7,62	2,16	3,53	19	T	A	G	T	A	C
UPLA 68	21,56	Long B	Jap	ARG	149	8,02	2,29	3,50	18	T	A	G	T	A	C
JEFFERSON	22,21	Long A	Jap	USA	157	6,79	2,44	2,78	20	G	C	G	T	A	C
SAFARI	22,37	Long A	Jap	PRT	149	6,60	2,72	2,43	17	G	C	T	T	A	C
A 301	22,44	Long B	Jap	USA	163	7,78	2,26	3,44	20	G	C	G	T	A	C
OTA	22,55	Long A	Jap	PRT	161	6,15	2,90	2,12	13	G	C	T	T	A	C
UPLA 64	22,57	Long B	Jap	ARG	148	7,87	2,16	3,64	17	G	C	T	T	A	C
LORD	22,60	Long A	Jap	ITA	144	6,93	2,60	2,67	20	G	C	G	T	A	C
SANDORA	22,75	Long A	Jap	HUN	135	7,39	2,62	2,82	17	G	C	T	T	A	C
DELLROSE	22,83	Long A	Jap	USA	163	6,58	2,27	2,90	20	G	C	G	T	A	C
BOMBA	22,84	Medium	Jap	ESP	159	5,45	2,99	1,82	21	G	C	T	T	A	C
N 3	22,86	Long A	Jap	ESP	167	6,64	2,58	2,57	20	G	C	G	T	A	C
KARNAK	22,90	Long A	Jap	ITA	156	7,45	3,43	2,17	17	G	C	T	T	A	C
GIGANTE VERCELLI	23,05	Long A	Jap	ITA	149	6,95	3,32	2,09	17	G	C	T	T	A	C
MARTA	23,06	Long B	Jap	ITA	183	7,40	2,29	3,23	20	G	C	G	T	A	C
COCODRIE	23,10	Long B	Jap	USA	155	7,01	2,10	3,34	20	G	A	G	T	A	C
DREW	23,26	Long B	Jap	USA	160	6,81	2,16	3,15	14	G	C	T	T	A	C
DELLMONT	23,33	Long B	Jap	USA	160	6,96	2,27	3,07	20	G	C	G	T	A	C
IR 5549-1-2	23,47	Long B	Jap	PHL	173	6,98	2,25	3,10	20	G	C	G	T	A	C
L 205	23,57	Long B	Jap	USA	156	7,47	2,21	3,38	10	G	A	T	C	G	T
BLUEBONNET	23,60	Long B	Jap	USA	-	8,16	2,66	3,07	20	G	C	G	T	A	C
TEJO	23,63	Long A	Jap	ITA	156	6,71	2,63	2,55	20	G	C	G	T	A	C
GOLFO	23,75	Long A	Jap	ITA	181	7,39	2,47	2,99	20	G	C	G	T	A	C
LAGRUE	23,76	Long A	Jap	USA	160	6,50	2,20	2,95	20	G	C	G	T	A	C
DORADO	23,79	Long B	Jap	GRC	164	7,29	2,23	3,27	14	G	C	T	T	A	C
PLUS	23,84	Long B	Jap	ITA	159	7,02	2,26	3,11	14	G	C	T	T	A	C
MAYBELLE	23,87	Long B	Jap	USA	155	6,65	2,19	3,04	20	G	C	G	T	A	C
L 204	23,90	Long B	Jap	USA	153	7,81	2,37	3,30	20	G	C	G	T	A	C
GIADA	23,93	Long B	Jap	ITA	151	7,45	2,08	3,58	17	G	C	T	T	A	C
BOND	23,96	Long B	Jap	USA	157	7,08	2,24	3,16	20	G	C	G	T	A	C
MERLE’	24,18	Long B	Jap	FRA	151	7,08	2,23	3,17	9	G	A	T	C	G	T
REXMONT	24,19	Long B	Jap	USA	161	6,72	2,19	3,07	10	G	A	T	C	G	T
LACASSINE	24,21	Long B	Jap	USA	162	7,03	2,21	3,18	20	G	C	G	T	A	C
ALAN	24,30	Long B	Jap	USA	154	6,86	2,03	3,38	14	G	A	T	T	A	C
DIXIEBELLE	24,35	Long A	Jap	USA	167	6,28	2,21	2,84	11	G	A	T	C	G	T
ARIANA	24,42	Long B	Jap	ROM	168	7,34	2,41	3,04	20	G	C	G	T	A	C
CNA 4081	24,44	Long B	Ind	BRA	149	6,64	2,19	3,03	9	G	A	T	C	G	T
A 201	24,50	Long B	Jap	USA	165	7,83	2,11	3,71	20	G	A	G	T	A	C
GLADIO	24,73	Long B	Jap	ITA	129	7,28	2,19	3,32	20	G	A	G	T	A	C
THAIBONNET	24,83	Long B	Jap	USA	159	7,81	2,37	3,30	20	G	A	G	T	A	C
ORIONE	24,85	Long A	Jap	ITA	173	6,90	2,65	2,60	9	G	A	T	C	G	T
IR 47686-9-4-1	25,03	Long B	Jap	PHL	172	7,14	2,19	3,26	20	G	A	G	T	A	C
FRAGRANCE	25,16	Long B	Jap	ITA	154	7,74	2,42	3,20	20	G	A	G	T	A	C
L.202	25,21	Long B	Jap	USA	155	7,52	2,18	3,45	20	G	A	G	T	A	C
ZHEN SHANG 47	25,40	Long A	Ind	CHN	152	6,10	2,92	2,09	11	G	A	G	C	G	T
ARROYOGRANDE	25,46	Long B	Jap	ESP	159	7,74	2,20	3,52	20	G	A	G	T	A	C
ALINANO C	26,03	Long A	Jap	FRA	151	7,38	2,57	2,87	11	G	A	G	C	G	T

The Apparent Amylose Content (AAC), with the EU classification, the group, the origin, the maturity time, the seed biometric characteristics and the alleles for the *GBSSI* molecular markers considered are indicated for each genotype.

^a^data are referred to decorticated grains.

### Molecular markers analyses

The germplasm collection of 127 rice accessions (Table 1) was chosen to evaluate the effectiveness of known molecular markers in predicting Apparent Amylose Content. Ten different alleles for the RM190 CTn microsatellite were identified (Table 2) but a clear relationship with the different AAC groups was observed only for some alleles (Table 1). Repeats CT_9_, CT_10_ and CT_14_ were present only in genotypes with 23–24.85% AAC, while CT_11_ and CT_20_ also identified accessions with AAC higher than 25%. Among this AAC classes, two varieties showed unique CT alleles: CT_13_ for Ota (22.55% AAC) and CT_21_ for Bomba (22.84% AAC). The most frequent allele, CT_18_ identified in 67 accessions, represents a wide range of AAC (14.92-21.56%). Similar results were observed for CT_17_ and CT_19_ which were associated to heterogeneous AAC intervals ranging from 15.55% to 23.27%. Considering non-glutinous genotypes, the RM190 microsatellite explained the 74.9% of the AAC variation in our collection (Table 3).

**Table 2:** Alleles identified for the RM190 microsatellite in the germplasm collection

**CT-repeats (RM190)**	**Allelic pattern SNPs intron 1 and exon 6**^**a**^	**Number of cvs.**	**AAC range (%)**	**AAC average (%)**
9	GA	3	24.18 – 24.85	24.49
10	GA	2	23.57 – 24.19	23.88
11	GA	3	24.35 – 26.03	25.26
13	GC	1	22.55	
14	GA	4	23.06 – 24.30	23.80
17	TA	6	16.42 – 23.27	19.05
17	GC	8	20.65 – 23.93	22.44
18	TA	67	14.92 – 21.56	18.37
19	TA	6	15.55 – 21.30	17.78
20	GA	7	23.10 – 25.46	24.71
20	GC	18	22.21 – 25.03	23.50
21	GC	1	22.84	

^a^The two bases indicate the SNP alleles in intron 1 and in exon 6, respectively.

**Table 3:** **Percentage of variation for Apparent Amylose Content (AAC) explained by the tested *GBSSI *** **molecular markers in the germplasm collection**

**Polymorphisms**	**Explained AAC variation (%)**	**p values**
CTn (RM190)	74.9	0.000
T/G intron1	77.5	0.000
A/C exon 6	30.9	0.000
CTn/intron1	77.7	0.260 CTn
		0.000 intron1
CTn/exon6	40.3	0.000 CTn
		0.000 exon6
Intron 1/exon6	79.5	0.000 intron1
		0.001 exon6
CTn/intron1/exon6	79.6	0.594 CTn
		0.000 intron1
		0.001 exon6
Intron1/exon6/-1,514	80.1	0.000 intron1
		0.001 exon6
		0.072 -1,514
CTn/intron1/exon6/-1,514	80.3	0.242 CTn
		0.000 intron1
		0.035 exon6
		0.038 -1,514
intron1/exon6/-1,514/exon9	80.1	0.000 intron1
		0.001 exon6
		0.073 -1,514
		0.965 exon9
CTn/intron1/exon6/-1,514/exon9	80.3	0.216 CTn
		0.000 intron1
		0.038 exon6
		0.035 -1,514
		0.675 exon9

For each marker, the explained variation and the associated p-value considering each molecular marker singularly or in association with others is reported.

For the SNP in the splicing site of the leader intron (intron 1), results confirming previous investigations were observed (Wang et al. 1995; Ayres et al. 1997; Bligh et al. 1998; Cai et al. 1998; Isshiki et al. 1998; Larking and Park 2003; Dobo et al. 2010). With only two exceptions represented by Rotundus and Antoni, all the accessions with AAC lower than 22% showed the T allele, while all the genotypes with AAC higher than 22% had the G allele (Table 1). This SNP explained a higher level of AAC variation (77.5%) with respect to the RM190 (Table 3) and the combination of the two polymorphisms did not significantly increase the level of explained variability (77.7%).

The presence of SNPs characterizing the different known *Wx* alleles was assessed in our collection through sequencing of exons 2, 4, 5 and 6. The *wx* allele was identified in the waxy cv. Calmochi 101, in which the 23 bp duplication (sequence motif: ACGGGTTCCAGGGCCTCAAGCCC) in exon 2 responsible for *Waxy* gene inactivation was present; literature data in fact provide AAC values for this cv. ranging from 0.8 to 1% (Park et al. 2007; Li et al. 2008). Allelic variation in the *Wx*^*in*^ allele, characterized by a non-conservative A/C mutation in exon 6, was observed with the A allele being detected, with few exceptions, in accessions with AAC ranging from waxy to 22% and higher than 24%, and the C allele generally present in genotypes with an AAC from 22 to 24% (Table 1). No polymorphisms were identified in exons 4 and 5, representative of the *Wx*^*mq*^ allele and, unlike in previous observations (Dobo et al. 2010), we did not observe mutations in exon 10 thus excluding the presence of SNPs in this exon.

Combining the results obtained for SNPs in intron 1 and exon 6, three allelic patterns were identified: GA, TA and GC (Table 2), the first letter indicating the G/T polymorphism in intron 1 while the second the A/C SNP in exon 6. Similarly to the behaviour observed for intron 1, most of the accessions from waxy to 22% AAC (with two exceptions) carried the TA haplotype (Table 1), GC pattern was present in accessions with 22-24% AAC (with two exceptions) and GA in accessions with AAC >24% (with three exceptions). Statistical analyses showed that the A/C SNP in exon 6 alone explained only the 30.9% of the variation in AAC, but altogether the two SNPs explained the 79.5%. Adding the RM190 microsatellite to the analysis, we did not find a significantly higher explanation of the AAC variation: 79.6% vs. 79.5% (Table 3).

The allelic variation at the RM190 locus together with intron 1/exon 6 SNPs data allowed the identification of allelic patterns associated to different AAC classes. In particular, even considering that no association of CT_17_ with a specific AAC group could be identified, all the accessions with haplotype CT_17_, G in intron 1 and C in exon 6 shared an AAC level ranging from 22 to 23%, with the exception of Giada (23.93%). Similarly, the CT_14_ allele associated with the CG and AG allelic patterns shared similar AAC (ranging from 23.2 to 23.8%). Among the CT_20_ group, the most frequent allelic pattern for high AAC class was CG, which identifies an AAC range from 22 to 24%, while in combination with AG frequently identified accessions with more than 25% AAC. CT_9_, CT_10_ and CT_11_ associated to the allelic pattern AG were typical of genotypes with more than 24% of AAC.

Despite the fact that the level of variation in AAC explained by our results is in agreement to the one recently evaluated in an Italian rice collection (Caffagni et al. 2013), it is consistently lower than previously observed. As examples, Ayres et al. (1997) using the combination of RM190 and the G/T in intron1 explained the 85.9% of the variation in AAC; Dobo and co-workers (2010) could explain the 93.8% of variation in AAC with RM190, the SNP in intron 1, the SNP in exon 6 and the SNP in exon 10 which was not present in our collection. Owing these results, it was realized that additional molecular markers could be needed to increase predictability of the different AAC classes within our germplasm panel.

### Allele mining of the *GBSSI* gene

**Figure 2:**
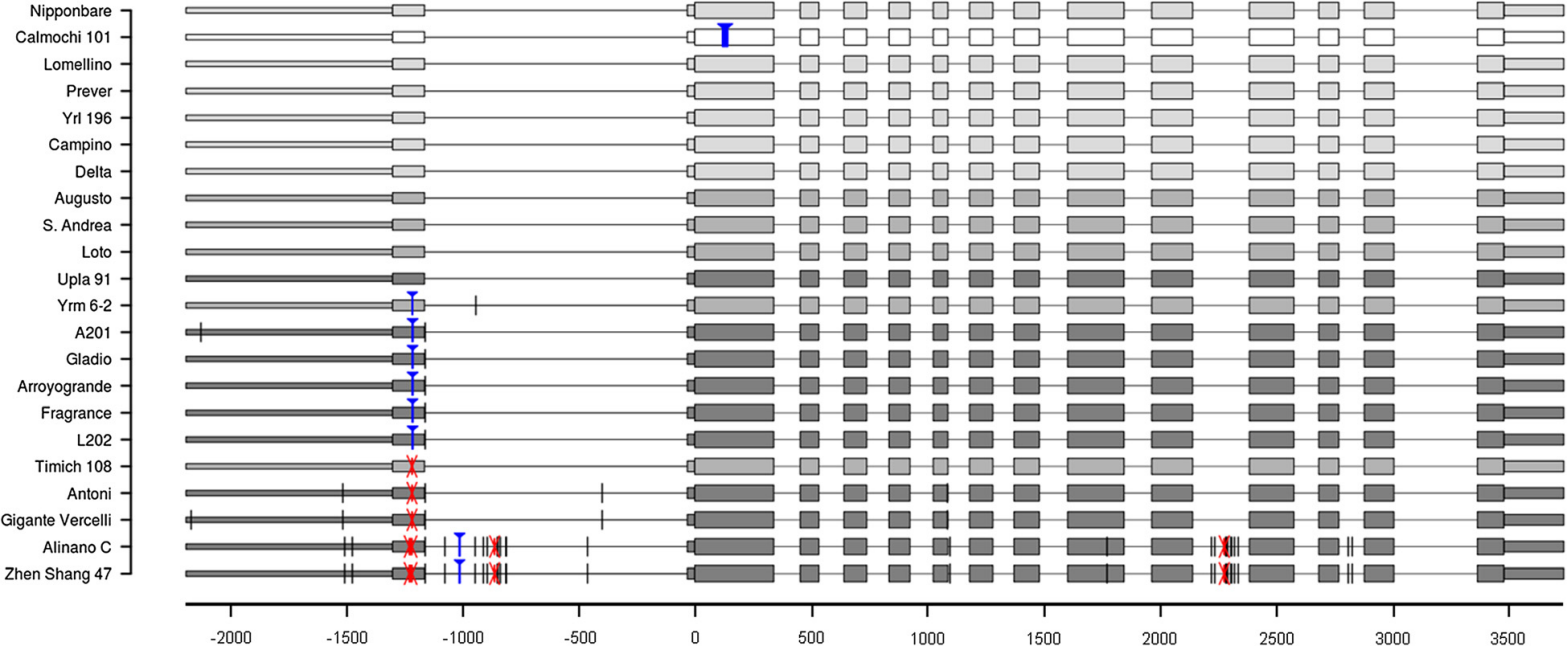
**Schematic representation of sequence alignment for the *GBSSI *alleles.** Nipponbare *Wx* sequence was used as reference. The position of the ATG is indicated with 0; negative numbers are referred to bp positions in the putative regulatory regions while positive numbers indicate bp positions in the coding region. Colour shade represents the different AAC classes from white corresponding to waxy to dark grey corresponding to high AAC. SNPs are symbolized by black bars, insertions by blue triangles and deletions by red crosses. Thick, intermediate and tight boxes indicate exons, 5′ and 3′ UTR and the promoter region, respectively. Lines between boxes represent introns.

To mine the genetic variation at the level of the *GBSSI* locus within our germplasm collection, the gene as well as 1kbp of the upstream putative regulatory region were sequenced in twenty-one genotypes representing all the AAC classes identified: Calmochi 101 for the waxy type; Prever, Yrl 196, Delta, Lomellino and Campino for 14-16% AAC; Yrm 6–2, Timich 108, Augusto, Loto and Sant’Andrea for 16-19% AAC; Upla 91, Antoni, Gigante Vercelli, A201 and Gladio for 21-25% AAC; and Fragrance, L 202, Zhen Shang 47, Arroyogrande and Alinano C for >25% AAC (Table 1). The twenty-one *GBSSI* sequences were compared by multiple alignments considering the Nipponbare sequence as the reference. Sequence comparisons showed an overall high level of similarity (Figure 2), indicating that the coding sequence was conserved in most of the genotypes with some exceptions and led to the identification of 32 SNPs (Table 4). The waxy cv. Calmochi 101 carried the *wx* allele with the 23 bp duplication in exon 2, as described before. Antoni and Gigante Vercelli accessions showed the non-conservative A/C SNP in exon 6, previously identified and causing a Serine/Tyrosine substitution in the GBSSI protein (Larking and Park, 2003). Additionally, these genotypes contained two common SNPs: one in the putative regulatory region (position −1,514) and one in the first intron (position −399). Gigante Vercelli also showed a SNP at −2,174 bp upstream the ATG (Figure 2; Table 4). Zhen Shang 47 and Alinano C carried a C/T SNP at position +1,801 in exon 9 which results in the substitution of Pro415 to Ser in the GBSS protein. Also for Zhen Shang 47 and Alinano C, SNP mutations were identified in the non-coding sequences: 2 in the promoter region, 11 in intron 1, 1 in intron 6, 9 in intron 10 and 2 in intron 12 (Figure 2; Table 4). Most of the SNPs identified in the present work are not classified in the group of SNPs computationally characterized by Kharabian (2010) and present in OryzaSNP (http://oryzasnp.plantbiology.msu.edu/cgi-bin/gbrowse/osa_snp_tigr/) and dbSNP (http://www.ncbi.nlm.nih.gov/sites/entrez?db=snp&TAbCmd=Limits) databases. Additionally to these SNPs polymorphisms, a 4 bp tandem repeat that was identified in rice cvs. showing high level of *Wx* transcripts (Cai et al. 1998) was highlighted at position +272 in the rice accessions Alinano C and Zhen Shang 47. In these two accessions, also a 1 bp deletion was present in intron 1 at position −862 and a 3 bp deletion in intron 10 at 2,241 bp from the start codon (data not shown).

**Table 4:** **SNPs identified from re-sequencing of the *Waxy *** **gene and the putative regulatory region**

**Genotype**	**AAC (%)**	**SNP**	**Position**	**OryzaSNP ID**
Yrm 6-2	16.40	G/A	-942	nc^a^
Gladio	24.73	T/G	-1164	nc
Arroyogrande	25.46	T/G	-1164	nc
Fragrance	25.16	T/G	-1164	nc
L202	25.21	T/G	-1164	nc
A201	24.50	A/G	-2132	nc
		T/G	-1164	nc
Antoni	21.27	G/T	-1514	nc
		T/G	-1164	nc
		G/T	-399	nc
		A/C	+1083	TBGI270316
Gigante Vercelli	23.05	T/A	-2174	nc
		G/T	-1514	nc
		T/G	-1164	nc
		G/T	-399	nc
		A/C	+1083	TBGI270316
Zhen Shang/Alinano C	25.40/26.03	A/G	-1522	nc
		C/T	-1481	nc
		T/G	-1164	nc
		G/A	-1079	nc
		A/G	-945	nc
		C/T	-918	nc
		A/G	-901	nc
		C/T	-850	nc
		A/C	-847	nc
		T/G	-837	nc
		C/T	-813	nc
		T/C	-811	nc
		T/C	-461	nc
		C/T	+1094	TBGI270317
		C/T	+1,801	nc
		G/A	+2218	nc
		G/A	+2232	nc
		A/G	+2279	nc
		G/A	+2282	nc
		T/C	+2288	nc
		G/A	+2301	nc
		T/C	+2305	nc
		C/G	+2317	nc
		A/G	+2333	nc
		C/T	+2806	nc
		G/A	+2823	TBGI270324

For each polymorphism, the rice accession where it was identified, the nucleotide mutation, the position with respect to the ATG and the ID in the OryzaSNP database are indicated.

^a^Not classified in the computationally characterized SNPs.

### Generation of new molecular markers from allele mining

**Figure 3:**
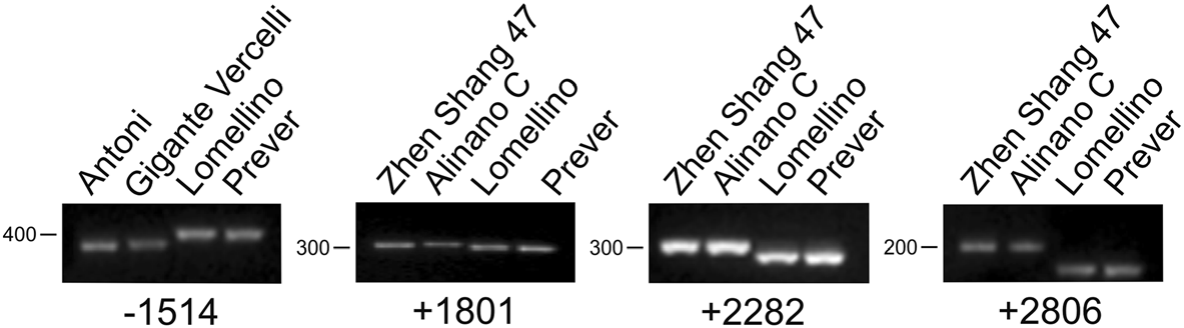
**dCAPS molecular markers developed from SNPs found in Antoni, Gigante Vercelli, Alinano C and Zhen Shang 47 *Waxy *alleles.** Lomellino and Prever were used as control. Primers and restriction enzymes for each molecular marker are listed in Additional file 1: Table S1.

With the aim of identifying more informative markers allowing a better discrimination between accessions with AAC higher than 25% from those with lower levels, one and three SNPs were selected from Antoni/Gigante Vercelli and Alinano C/Zhen Shang 47, respectively, for molecular markers development. dCAPS molecular markers were obtained from the SNPs identified at positions −1,514 (promoter region) (Antoni and Gigante Vercelli), +1,801 (exon 9), +2,282 (intron 10) and +2,806 (intron 12) (Alinano C and Zhen Shang 47) and used to screen the 127 accessions (Figure 3; Table 1). Antoni and Gigante Vercelli haplotype (T) at position −1,514 was identified in 17 additional accessions belonging to the group with AAC 20.65 – 24.85%. Among them, 11 carried the C allele for the SNP in exon 6 as Antoni and Gigante Vercelli. Considering both the SNPs, four allelic patterns associated to different levels of AAC were identified (Table 5). The AG allele (the first base referred to the SNP in exon 6, while the second to position −1,514) was present in 89 accessions and associated to a mean AAC value of 18.99% (without considering the waxy variety Calmochi 101); the GC allele was found in 18 accessions with a mean AAC value of 23.50%; the AT allele, carried by 6 accessions, corresponded to an AAC average of 24.24%; the last allele, CT, was identified in 13 genotypes with a mean AAC of 22.75%.

**Table 5:** Apparent Amylose Content (AAC) values observed in accessions with different allelic patterns for the newly identified SNP at position −1,514 in Antoni and Gigante Vercelli associated to the exon 6 SNP

**Allelic pattern**^**a**^	**Number of accessions**	**AAC range (%)**	**Mean of AAC (%)**
GA	89	14.92 – 23.27	18.99
GC	18	22.21 – 25.03	23.50
TA	6	23.57 – 24.85	24.24
TC	13	20.65 – 23.93	22.75

^a^The two bases indicate the SNP alleles at position -1,514 and in exon 6, respectively.

Considering the RM190 alleles associated to the four haplotypes identified for exon 6 and the SNP at −1,514, it was observed that the combination of CT_20_ with A (exon 6) and G (SNP −1,514) was always associated to an AAC higher than 24.5% (Table 1), thus providing a previously un-indentified tool for selecting rice accessions with high AAC. Allelic variation observed for the SNPs at positions +1,801, +2,282 and +2,806 can finally provide useful diagnostic tools for selecting accessions with AAC higher than 24% when specific donors like Merlè, CNA 4081, Orione, Zhen Shang 47 and Alinano C are used (Table 1); for these accessions, in fact, a unique CGT allelic pattern was observed for the three SNPs. The SNP at position −1,514 slightly increased the AAC explained variation to 80.1% when considering the SNPs only, and to 80.3% when the RM190 was included (Table 3). However, when only the models having all the variables significant at P ≤ 0.05 were considered, the model with the highest ability to explain AAC variation (79.5%) was the one with SNPs at intron1 and exon 6 (Table 3), a result which is fully in agreement with previous work (Caffagni et al. 2013).

### Relationships between grain shape parameters and *Waxy* haplotypes

**Figure 4:**
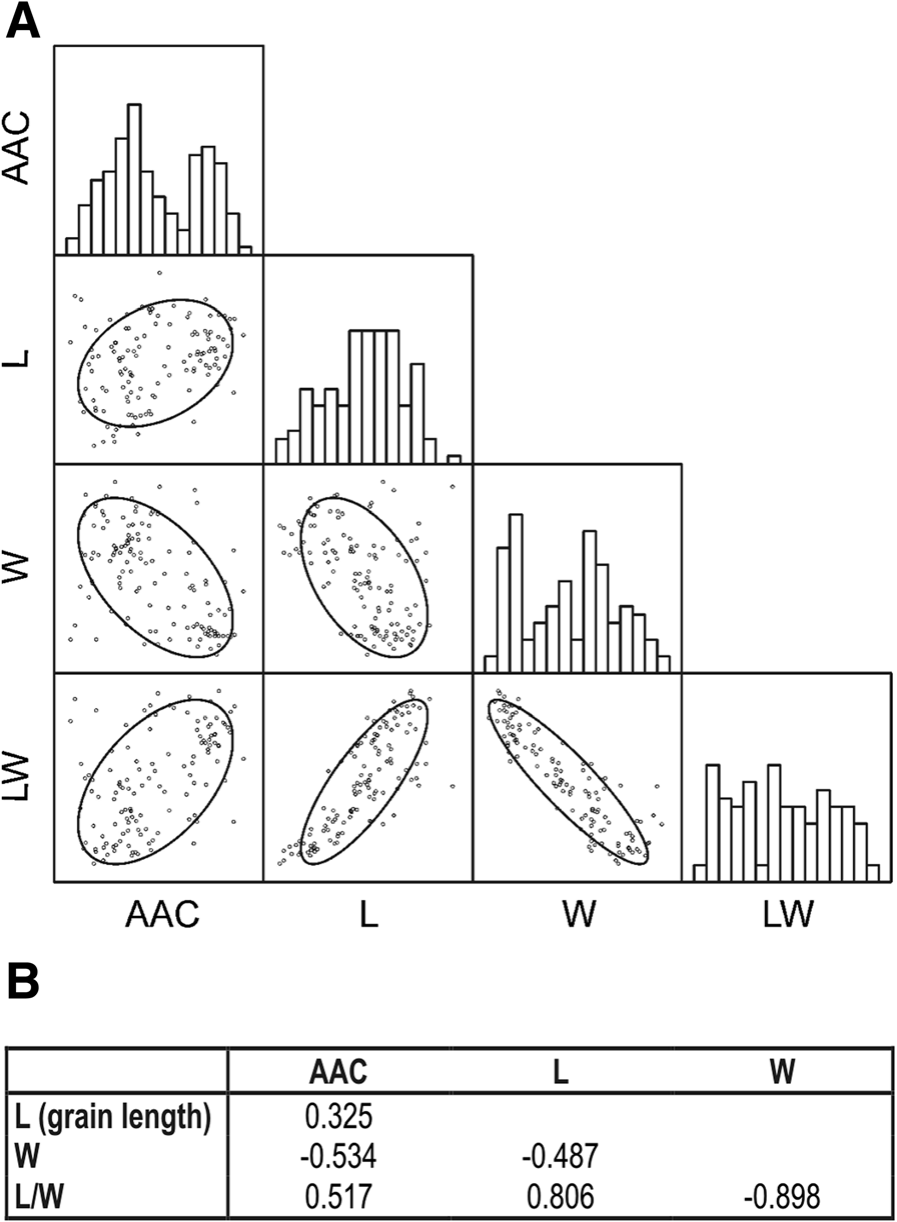
**Correlations between grain shape parameters and AAC. A)** Scatterplot matrix of grain (dehulled caryopses, i.e., brown rice) characters. Histogram distribution plots for the single variables are shown on the diagonal cells. Below them the bivariate distributions are shown for every pair of traits. Confidence ellipses (ELL) mark confidence limit for each distribution (P = 0.95, for a normal bivariate); **B)** Pearson’s correlation coefficients for grain (dehulled caryopses, i.e., brown rice) characters. All the correlations are significant with P ≤ 0.001 (probabilities were adjusted by Bonferroni’s correction for multiple tests; n = 126).

Correlation analyses between grain shape parameters for the 126 non-glutinous rice accessions (Table 1) are indicated in Figure 4 A, B and showed a close agreement with those reported by Tran et al. (2012). The most obvious correlations were between the L/W ratio and either grain length (positive) or grain width (negative). A negative correlation exists between the length and the width of the grain while regarding AAC, grain length and the L/W ratio were positively correlated with it, whereas grain width was negatively correlated. An analysis of the histogram distribution plots of the variables indicated that the distributions of AAC and grain width clearly showed two modes (Figure 4 A).

In rice, traits related to grain size, shape and cooking properties have a large impact on market appreciation and play a pivotal role in the adoption of new varieties (Webb 1991; Juliano 2003). It is therefore interesting to note that, as shown in Table 6, we found some surprising associations between the haplotypes at the *waxy* locus and the shape of the rice caryopsis. In particular, the SNP at the first intron (which identifies *Wx*^*a*^ and *Wx*^*b*^ genotypes) was associated with differences in the width of the grain, the length to width ratio (L/W), and the length of the kernel. The SNP at the sixth intron showed no association with these traits by itself, but it slightly increased the overall explained variance once the SNP at the first intron was considered. This suggests that the latter SNP was the actual responsible of the association, whereas the SNP at the intron 6 has only an ancillary effect. Even the CT_n_ showed a significant association, but its explanatory capability of the variance of grain biometric parameters was lower than that of the SNP at the first intron, thus that, given it also has no direct effect on the functionality of GBSSI, its association is most probably indirect and most likely ascribable to the phylogenetic association of some CT_n_ haplotypes with the two versions of the SNP at the first intron.

**Table 6:** **Percentage of variation for seed biometric indexes explained by the tested *GBSSI *** **molecular markers in the germplasm collection**

	**Decorticated grain lenght**		**Decorticated grain width**		**Lenght to width ratio**	
**Molecular marker**	**Variation (%)**	**p-value**	**Variation (%)**	**p-value**	**Variation (%)**	**p-value**
SNP intron1	30.8	0.000	49.7	0.000	47.7	0.000
CTn	10.8	0.136	37.7	0.000	30.2	0.000
SNP intron1/SNP exon6	30.9	0.000 intron1	52	0.000 intron1	49.3	0.000 intron1
		0.791 exon6		0.051 exon6		0.11 exon6
SNP intron1/CTn	21.8	0.043 CTn	37.7	0.008 CTn	33.7	0.033 CTn
		0.000 intron1		0.798 intron1		0.015 intron1
SNP intron1/CTn/SNP exon6	22.8	0.000 intron1	38.3	0.41 intron1	35.4	0.003 intron1
		0.029 CTn		0.020 CTn		0.029 CTn
		0.243 exon6		0.295 exon6		0.087 exon6
SNP intron1/SNP exon6/SNP intron6	10.2	0.007 intron1	29.2	0.000 intron1	26.0	0.000 intron1
		0.779 exon6		0.045 exon6		0.102 exon6
		0.363 intron6		0.055 intron6		0.098 intron6
SNP intron1/SNP exon6/SNP intron6/CTn	22.9	0.019 intron1	39.3	0.465 intron1	35.6	0.316 intron1
		0.244 exon6		0.293 exon6		0.088 exon6
		0.627 intron6		0.188 intron6		0.566 intron6
		0.037 CTn		0.037 CTn		0.064 CTn

For each marker the explained variation and the associated p-value considering each molecular marker singularly or in association with others are reported.

**Figure 5:**
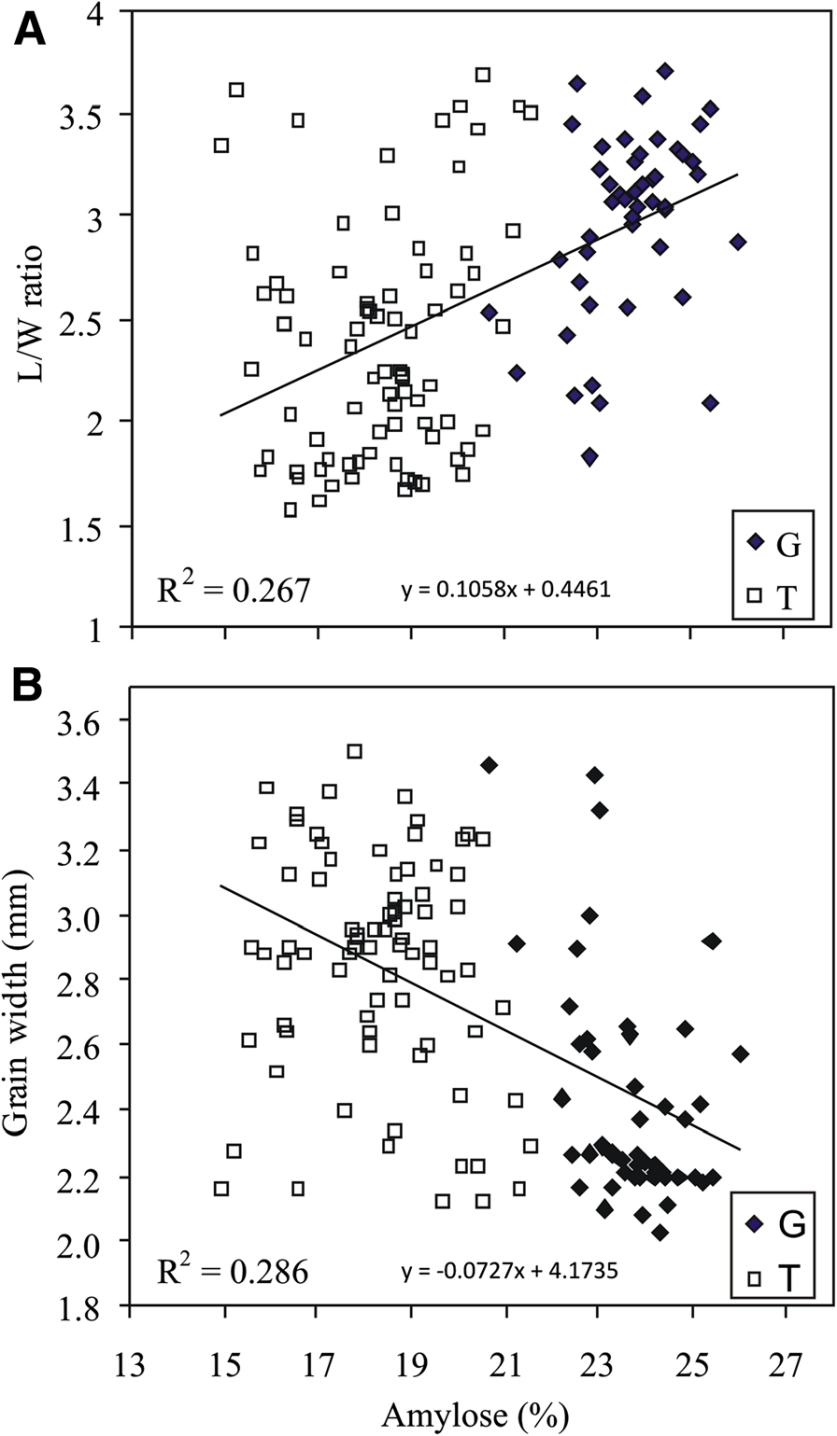
**Relationships between AAC and L/W ratio correlations.** Scatterplots of: L/W ratio *versus* AAC **(A)**, and grain width *versus* AAC **(B)**. Datapoints for dehulled caryopses of 126 non glutinous cultivars, grouped according to the haplotype at the SNP of intron 1 (47 accessions with G allele; 79 accessions with T allele). The regression line for the whole dataset and its determination coefficient are shown in each plot.

The positive correlation between AAC and L/W ratio, and the negative one with the width of the grain were further analysed (Figure 5). In fact, by plotting L/W ratio *versus* AAC for the overall genotype set used in this work, the positive correlation (r = 0.517, P < 0.001) is immediately apparent (Figure 5A). When the genotype set is grouped according to the haplotype at the SNP at intron 1 (G/T), that is, the *Wx*^*a*^/*Wx*^*b*^ allelic version is superimposed onto the correlation plot, it becomes evident that: (a)- this SNP offers a sharp distinction between genotypes with AAC ≤ 21% (*Wx*^*b*^) and AAC ≥ 21% (*Wx*^*a*^); (b)- in our set of genotypes, there seems to be a break for AAC around 21.5%, which, therefore, appears to be a more precise threshold value for discriminating between *Wx*^*a*^/*Wx*^*b*^ allelic versions, even though a few genotypes spill over, and small differences in AAC can actually occur because of the method of assay (Fitzgerald et al. 2009); (c)- the frequencies of genotypes having an intermediate Apparent Amylose Content (haplotype *Wx*^*b*^) are skewed towards low L/W ratios, and, *vice-versa*, the frequencies of high amylose genotypes (haplotype *Wx*^*a*^ ) are skewed towards high L/W ratios. It is indeed the presence of these skewed distributions that generates the positive correlation between L/W ratio and AAC, and, then, the association between L/W ratio and the SNP at the first intron.

Furthermore, the inverse correlation between AAC and grain width is slightly stronger than that between AAC and L/W (Figure 4; Tran et al. 2012). Correspondingly, grain width has a slightly higher explanatory ability on AAC than L/W ratio (Figure 5B). This is due to the stronger skewing of the grain width distribution within the *Wx*^*a*^ haplotype, which, in turn, is linked to the up-mentioned presence of two clearly distinct modes in grain width (Figure 4A): one, at 2.2 mm, typifies *Wx*^*a*^ genotypes, while the other, around 3 mm, characterizes *Wx*^*b*^ genotypes (Figure 5B). In the case of width, *Wx*^*a*^ genotypes are more sharply crowded close to their mode than what occurs for the L/W ratio, that is, their frequencies are more skewed towards thinner grains. This makes the existence of two different modes, one for each *Wx* haplotype, immediately evident even in the overall population (Figure 4A), and also augments the slope of the regression line (Figure 5B), thus that the effect of the *Wx* haplotype upon grain width appears to be greater than that on L/W ratio.

Interestingly, AAC also positively correlates with cycle length (r = 0.378, P < 0.001; data for 124 non-glutinous rice accessions for which maturity data were available; Table 1), whereas no significant correlation was observed between cycle length (days to maturity) and grain shape parameters (data not shown). The relationship would appear to be owed to a preponderance of long-cycle accessions in the high-amylose *Wx*^*a*^ group (data not shown). It is however not a necessary correlation, since some genotypes with high AAC and short cycle were present as well (Gladio and Sandora; Table 1). For the same reason, this should not be an environmentally-caused relationship, although the amylose content can be affected by the temperature during grain ripening (Gomez 1979).

## Discussion

### Allelic analyses of the *Waxy* gene

Different alleles of the *Waxy* gene in rice are defined by a relatively small number of molecular markers. The RM190 CT_n_ itself could explain more than 80% of Apparent Amylose Content variation in several different rice collections (Shu et al. 1999; Bergman et al. 2001; Tan and Zhang 2001; Dobo et al. 2010). According to other works, for our germplasm collection RM190 could explain a high percentage of variation in AAC (74.5%), considering non-glutinous accessions. Dobo and co-workers (2010) found that the CT_20_ allele was associated to high or intermediate AAC for European or US cvs., respectively; similarly, in our analyses, this allele was always associated to AAC higher than 22% but regardless to the origin of the genotype. As previously observed (Bergman et al. 2001; Tan and Zhang 2001; Dobo et al. 2010), accessions with a number of CT repeats ranging from 17 to 19 showed an AAC variable from 15 to 23%. This behaviour is in agreement to the fact that the RM190 microsatellite is only a molecular marker associated to the *Waxy* gene without a functional role in determining the level of amylose in rice seeds (Chen et al. 2008; Dobo et al. 2010).

A functional role was instead characterized for the G/T SNP in the donor splicing site of the leader intron. In literature the G haplotype, which characterises the *Wx*^*a*^ allele, is associated to high AAC, while the T allele (*Wx*^*b*^) to low and intermediate AAC (Shu et al. 1999; Bergman et al. 2001; Tan and Zhang 2001; Dobo et al. 2010). For our non-glutinous germplasm, the G haplotype was always associated to AAC higher than 22%. Considering the high level of AAC variation explained (77.5%) and the observation that only a very small increase in explained AAC variation resulted from adding the RM190 data, we concluded that our results are in accordance with the SNP in intron 1 as being the main determinant affecting amylose amount in endosperm.

Several researches have been focused in identifying different *Wx* alleles associated to the wide variability in AAC. Larkin and Park (2003) found two SNPs, an A/C in exon 6 and a C/T in exon 10, causing non-conservative amino acid substitutions at the protein level which could change the specific activity of the GBSSI protein. Together with the SNP in intron 1, these polymorphisms explained 93.8% of ACC variation in European and US germplasm collections (Dobo et al. 2010). Chen et al. (2008) screened 171 rice accessions originating from 43 countries for the above mentioned *GBSSI* SNPs and found that the majority of cvs. with an AAC ranging from 21 to 22% carried the haplotype C at exon 6. Results with our collection highlighted no variations for exon 10 and an association of the SNP in exon 6 to AAC values from 21 to 24% for the C allele, while the A allele was present in accessions with AAC ranging from waxy to 21% and from 24 to 26%. The three haplotypes defined by the combination of the two SNPs in intron 1 and exon 6 were able to explain the 79.5% of AAC variation in non-glutinous accessions. Even in this case, when the RM190 was added to statistical analyses it did not show a significant effect on the percentage of explained AAC variation, confirming that this molecular marker does not have a direct effect on the functionality of the enzyme (Dobo et al., 2010). These results underline that the two SNPs (intron 1 and exon 6) represent the more suitable tools to be used in breeding programs for our germplasm collection and that *a priori* generalization of the efficiency for molecular markers in predicting AAC values could represent a risky procedure.

A higher level of AAC variation was found to be explained in previous work with respect to the ones observed here, even when comparisons were based on the same molecular markers. Considering that our collection is represented by genotypes originated from a wide range of countries, it is possible that the genetic diversity to be explained was greater than in other studies where the germplasm originated from more restricted areas: US (Ayres et al. 1997; Bergman et al. 2001), Europe and US (Dobo et al. 2010), China (Tan and Zhang 2001) and Korea (Shu et al. 1999).

### Identification of new molecular markers associated to apparent Amylose content

To date, most of the studies on the topic have been based on the characterization of molecular markers associated to waxy and low AAC (Isshiki et al. 1998; Larkin and Park 2003; Wanchana et al. 2003; Mikami et al. 2008; Liu et al. 2009; Tran et al. 2012). However the preference for rice with high AAC in occidental countries underlines the need of developing new markers facilitating the identification of genotypes with high AAC. A sub-sample of 21 genotypes belonging to our collection and representing four AAC classes was selected for the *GBSSI* gene re-sequencing. A very high level of sequence identity was observed and most of the identified polymorphisms were detected in non-coding regions. Thus, the *GBSSI* gene has been likely conserved during evolution as its key role in controlling the accumulation of amylose in caryopsis.

As previously reported, the amount of amylose in endosperm is principally related to the post-transcriptional regulation of the *Waxy* gene which is influenced by the intron 1 SNP (Wang et al. 1995; Bligh et al. 1998; Cai et al. 1998). However, our sequencing comparisons revealed that genotypes belonging to different AAC classes showed the same *GBSSI* haplotypes and alleles suggesting that other genes involved in starch synthesis can affect the amylose content in endosperm. These additional genes could be represented by the *Du* gene, encoding for a Starch Synthase enzyme (Zeng et al. 2007), the *Shr* gene encoding for a Sucrose Synthase (Kawagoe et al. 2005), or other genes encoding for enzymes involved in starch metabolism such as ADP glucose pyrophosphorylase (Venu et al. 2011) and α-glucan phosphorylase (Satoh et al. 2008).

Antoni and Gigante Vercelli accessions carried the SNP in exon 6 typical of the *Wx*^*in*^ allele, the SNP in intron 1 (position −399) and one in the promoter region (position −1,514). Moreover, two accessions with high AAC, Zhen Shang 47 and Alinano C, showed the highest sequence variation with respect to Nipponbare that included a non-conservative SNP in the coding region of exon 9, causing a Pro to Ser substitution in the GBSSI protein. As this mutation implicates the substitution of an apolar amino acid to a polar one it should be possible that it increases the activity of the GBSSI enzyme, even if its effect should be small as the SNP does not affect the AAC variation significantly (Tab. 3).

New dCAPS markers were developed exploiting the SNPs in intron 1 at position −1,514 (identified in Antoni and Gigante Vercelli), exon 9, intron 10 and intron 12 (identified in Zhen Shang 47 and Alinano C). After screening of the 127 accessions with these molecular markers it was observed that the combination of the CT_20_ RM190 alleles in combination with the A haplotype for exon 6 and the G haplotype for SNP −1,514 was always associated to an AAC higher than 24.5% thus providing an efficient tool for selecting high AAC rice accessions.

### Associations between molecular markers and grain shape parameters

The results presented in this work confirm general observations (Webb 1991; Juliano 2003) that high AAC is associated with a slender grain, whereas rices having an intermediate amylose content most often have bold grains. We clearly highlighted that the frequencies of genotypes having a low AAC (< 21.5%) are skewed towards low L/W ratios while the frequencies of intermediate-high amylose genotypes (>21.5% AAC) are skewed towards high L/W ratios. These skewings can be imputed to selection for consumer preferences: for cooking and processing, rice are conventionally classified as short-, medium- and long-grain types, and an AAC ≥ 20% is preferred for long-grain rice, whereas an AAC < 20% is commonly favoured for short- and medium-grain types (Webb 1991). The width of the grain is as much, or even more, linked to the cooking application of rice than the L/W ratio: long, thin-grain rice is typically used in applications requiring distinct shape and texture, and high amylose ensures that cooked grains are firm and remain separate; on the other hand, medium- and short-grain rice are ideal for puddings, desserts, and similar applications, and low amylose content allows to obtain cooked grains that are soft, moist and sticky. Grain width and L/W ratio are more distinctive of the cooking application than grain length because long-grain rice with bold kernel are typically used to prepare ‘risotto’, and many of them are traditional varieties with intermediate AAC. In fact, the EU classification (see Ferrero and Nguyen, 2004 for a synoptic table of USA and EU grades) further distinguishes long-grain rice (length > 6.0 mm) into long A (2 < L/W < 3) and long B types (L/W ≥ 3). The former wide-grain type is used for ‘risotto’ and often has intermediate AAC, while the latter thin-grain type includes high amylose rice for oriental and side dishes, prepared entrees, rice salad and garnitures. Thus, grain width is more related to cooking applications than grain length, and then it has a better association with AAC. Further work is crucial to characterize the genetic basis of the two different modes observed for grain width in *Wx*^*a*^ and *Wx*^*b*^ haplotypes, and many candidate genes are available (Huang et al. 2013).

It can be worthy to note that the positive correlation between AAC and cycle length may occur since the high-amylose *Wx*^*a*^ haplotype, primarily found in the *indica* subspecies (Hirano and Sano 1991; Dobo et al. 2010), was most probably introduced into the *japonica* subspecies, which till to some decades ago lacked cultivars with high amylose (Juliano 1979), from indica genotypes, which frequently have longer cycles. It can be supposed that breeding for high AAC japonica cultivars has then been commonly done by crossing within a restrict group of late-maturing materials of indica derivation, thus that the long cycle would be an instance of genetic drift (or draft). On the other hand, the fact that no significant correlation was observed between cycle length and grain shape parameters suggests that breeding for both early and late cultivars within each of the grain-type varietal groups has broken up any hitchhiking effect between these traits. The strong association between the SNP at the first intron (which identifies *Wx*^*a*^ and *Wx*^*b*^ genotypes) and the width of the grain, the length to width ratio (L/W), and the length of the kernel is owed to the skewing of genotype frequencies towards a bold grain in *Wx*^*a*^ rice and towards a slender grain in *Wx*^*b*^ ones (Figure 5), and hence it is most likely ascribable to human selection. Simultaneous selection for two traits can increase the frequency of alleles that affect both traits favourably and leave the frequency of alleles that affect one trait favourably and one trait unfavourably at lower levels (Bennett and Swiger 1980), which is precisely what is observed in Figure 5. Selection for optimal character combinations, generating genetic correlation between suites of linked traits, is also known as correlational selection (Sinervo and Svensson 2002). Together with selection for non-shattering grains, reduced seed dormancy, whitish kernels and aroma (McCouch et al. 2012; Sang and Ge 2013), correlational selection for grain shape and AAC has marked the evolution of the rice crop according to human preferences for grain characteristics.

## Conclusions

The available molecular markers utilized for evaluation of AAC classes did not provide tools for predicting accessions with AAC higher than 24.5%. New SNPs were identified through re-sequencing of the *Waxy* gene and 1kbp of the upstream region. The related dCAPS markers increased the AAC explained variation and allowed the identification of a haplotype almost unequivocally associated to AAC higher than 24.5%, which represent the AAC class preferred in the occidental countries markets. The SNP at the first intron, which identifies the *Wx*^*a*^ and *Wx*^*b*^ alleles, was associated with differences in the width of the grain, the L/W ratio and the length of the kernel, most likely as a result of human selection.

## Methods

### Plant material and phenotyping for AAC and biometric parameters

An Italian *Oryza sativa* L. collection of 127 accessions was used in the present study and included 40 national rice varieties belonging to the *japonica* ssp. and 87 foreign accessions (6 from Spain, 6 from France, 29 from the US, 10 from Portugal, 7 from Argentina and 29 from other countries worldwide), of which 125 were *japonica* and 2 *indica*. The Italian rice varieties were selected because of their past and present relevance for breeding programs, and their contribution to rice production in the last decades. The 87 foreign accessions were selected as being relevant to Italian rice breeding, or considered reference varieties at the international level.

Each rice accession was grown in triplicate field trials for two years. Maturity time of each field trials was recorded when at least 50% of the plants was ready for harvesting. Data was represented by the mean value of three replicates and indicates the days from sowing to panicles maturation. Harvested rice seeds were used for amylose quantification and biometric parameters evaluations. According to the protocol of Williams et al. (1958), with modifications by Inatsu (1988), the AAC of milled grain was measured with a FOSS FIAstar 5000 auto-analyzer which is based on a flow injection of a solution of NaOH 0.09% to the sample, the addition of an iodine solution and the spectrometric determination of the absorbance of the formed color at 720 nm. The calibration was performed measuring the absorbance of standard rice samples carrying 15.40%, 23.10% and 27.7% of AAC, respectively, using a white reference. These reference samples were supplied and certified by FOSS as having their amylose content determined against amylose/amylopectin standards. The SoFIA software (FOSS) was used to build up the calibration curve and to obtain the percentage of amylose in our samples. Each reference analysis was repeated twice for each sample. Biometric parameters of rice seeds (decorticated grain length and decorticated grain width) were evaluated trough optical scanner-produced high resolution images analyzed with the WinSEEDLE 2011a software (Regent Instruments Inc.).

### Molecular markers analyses

To obtain genomic DNA from the rice accessions, seeds were germinated in petri dishes at 30°C and one-week old seedlings were transplanted and grown in a greenhouse until three leaf stages; leaves were therefore collected, frozen in liquid nitrogen and store at −80°C. Genomic DNA was extracted on plates using the Wizard® Magnetic 96 DNA Plant System (Promega) according to manufacturer’s instructions. The CTAB DNA extraction method (Doyle and Doyle 1987) was instead applied for the 21 genotypes selected for *GBSSI* gene re-sequencing.

The RM190 CT repeat was assayed using the M13-tailed forward primer RM-190 F (CACGACGTTGTAAAACGACCTTTGTCTATCTCAAGACAC) and the reverse primer RM-190R (TTGCAGATGTTCTTCCTGATG) (Ayres et al. 1997; Chen et al. 2008). PCR reactions were performed in 10 μl containing 15 ng of genomic DNA, 0.1 μM of RM-190 F, 1 μM of RM-190R and FAM-labelled M13 (CACGACGTTGTAAAACGAC), 0.2 mM dNTPs and 1 U GoTaq DNA Polymerase (Promega). DNA was amplified using a touchdown program as follows: denaturation at 94°C per 3 min, 20 cycles at 94°C per 45 sec, from 61°C to 51.5°C per 45 sec, reducing the annealing temperature of 0.5°C for each cycle, and 72°C per 45 sec, 24 cycles at 94°C per 45 sec, 51°C per 45 sec and 72°C per 45 sec and a final extension of 72°C per 10 min. Labelled PCR products were run in a 3130 Genetic Analyzer (Applied Biosystems). ROX size standard (Applied Biosystems) was used.

PCRs for dCAPS analyses of the G/T polymorphism in intron 1 were carried out in 20 μl using GoTaq DNA Polymerase (Promega) supplemented with 5% DMSO and with 20 ng of genomic DNA, 1 μM of GBSS-W2F, 1 μM of GBSS-W2R (Ayres et al. 1997; Chen et al. 2008). Amplification conditions were: 94°C per 4 min followed by 40 cycles of 94°C per 40 sec, 60°C per 50 sec and 72°C per 1 min per kb and a final extension of 72°C per 10 min. After amplification 5 μl of PCR products were digested with 1 U of *AccI* restriction enzyme (New England BioLabs) in a total volume of 10 μl at 37°C over night. The samples were run on 2% agarose gel. Each digested sample was compared alongside with its not-digested cognate control.

The presence of *Wx*^*op*^, *Wx*^*in*^, *Wx*^*mq*^, *Wx*^*hp*^ and *wx* alleles and the variability for the SNP in exon 10 identified by Larking and Park (2003) were assessed in our collection through the sequencing of exons 4, 6, 5, 2 and 10 using the same procedure for the allele mining experiment described below.

To detect the SNPs at position −1,514, +1,801, +2,282 and +2,806 by dCAPS analysis, mismatched forward primers were designed by dCAPS Finder 2.0 software (http://helix.wustl.edu/dcaps/dcaps.html). Primers and restriction enzymes for dCAPS assay are listed in Additional file 1: Table S1. For PCR amplification, the same protocol used for detecting the G/T polymorphism in the leader intron was used.

### Allele mining

For the allele mining of the *GBSSI* alleles in the 21 selected genotypes, six overlapping regions, ranging from 800 bp to 2,500 bp, were PCR amplified from genomic DNA with the same protocol described above for the G/T polymorphism. Primers designed on the Nipponbare genomic sequence (Genebank AC NC_008399; Additional file 2: Table S2) were utilized for genomic DNA amplifications using the combinations indicated in Additional file 2: Table S2. The amplified regions covered the entire gene plus 1 kbp of the upstream putative regulatory region. After gel purification by the Wizard® SV Gel and PCR Clean-Up System (Promega), PCR products were directly sequenced. Sequencing reactions were accomplished by the use of ABI BigDye Terminator version 3.1 (Applied Biosystem) in forward and reverse directions with 5 μl of each PCR amplification product and the same primers used for PCR amplifications or internal primers. All primers were designed using the Primer3 0.4.0 software (http://frodo.wi.mit.edu/) and blasted against the rice genomic sequence on the Gramene website (http://www.gramene.org) to ensure the specificity for the *GBSSI* gene.

### Computational analyses

The RM190 microsatellite was analyzed using the GeneMapper software (Applied Biosystem). Sequence assembly was assessed with the ContigExpress tool of Vector NTI Software (Invitrogen) using the Nipponbare genomic sequence as reference. Sequence comparison was carried out by MultAlin software (http://multalin.toulouse.inra.fr/multalin/). All data were analysed with the Systat 12 software (SPSS Inc., Chicago, IL, USA). The relationships between numerical variables (AAC and grain biometrical characters) were evaluated by Pearson correlation coefficients and regression analysis. The associations between numerical variables (AAC) and categorical variables (marker haplotypes) were analysed according to the General Linear Model (GLM) procedure.

## Competing interests

The authors declare that they have no competing interests.

## Authors’ contributions

CB carried out the molecular genetic studies, the sequence alignment and drafted the manuscript; DC, RP, PB, SU, GO, FD participated to molecular genetic studies, the sequence alignment and determination of Apparent Amylose Content; AG participated in data analysis and helped to draft the manuscript; EL, LC participated in the design of the study and helped to draft the manuscript; GV designed the study and helped to draft the manuscript. All authors read and approved the final manuscript.

## Supplementary Material

Additional file 1: Table S1 Markers obtained from the newly discovered SNPs. For each marker, primers, SNP position, restriction enzyme and origin of the restricted amplicon are indicated. The mutated base is in bold and underlined. Click here for file

Additional file 2: Table S2 Primer combinations used to amplify the Waxy alleles and internal primers used for amplicons sequencing. Click here for file
