# Supplementary material for: Improvement of marker-based predictability of Apparent Amylose Content in japonica rice through GBSSI allele mining
Source: Rice (N Y). 2014 Jan 2;7(1):1. doi: 10.1186/1939-8433-7-1 (PMC3904453; doi:10.1186/1939-8433-7-1)
Supplement: Additional file 1: Table S1 — Markers obtained from the newly discovered SNPs. For each marker, primers, SNP position, restriction enzyme and origin of the restricted amplicon are indicated. The mutated base is in bold and underlined. [file 1939-8433-7-1-S1.docx]

Table S1. Markers obtained from the newly discovered SNPs. For each marker, primers, SNP position, restriction enzyme and origin of the restricted amplicon are indicated. The mutated base is in bold and underlined

| **SNP position with respect to ATG** | **Marker name** | **Primer forward** | **Primer reverse** | **Restriction enzyme** | **Digested allele** |
| --- | --- | --- | --- | --- | --- |
| -1,514 | dCAPS-1514 | TCTCTTCTCTCTCCCGTCCCGT**A**GC | CACAAGCAGAGAAGTGAAGCA | *AluI* | mutant |
| +1,801 | dCAPS+1801 | GCATGGACGTCAGCGAGTGGGAT**G**C | CTTCCGGCTAACTCCACAAG | *NheI* | wild type |
| +2,282 | dCAPS+2282 | AGAAGGGGTGAGGCTTTGAACCC**C**G | CCCTGCAGCTGGATGAGT | *PvuI* | wild type |
| +2,806 | dCAPS+2806 | TACATAACAATCAGATATGACAC**C**T | TTCGTAATTTATACCTTCCAGGAGA | *MnlI* | wild type |
