# Supplementary material for: Improvement of marker-based predictability of Apparent Amylose Content in japonica rice through GBSSI allele mining
Source: Rice (N Y). 2014 Jan 2;7(1):1. doi: 10.1186/1939-8433-7-1 (PMC3904453; doi:10.1186/1939-8433-7-1)
Supplement: Additional file 2: Table S2 — Primer combinations used to amplify the Waxy alleles and internal primers used for amplicons sequencing. [file 1939-8433-7-1-S2.doc]

**Table S2** Primer combinations used to amplify the Waxy alleles and internal primers used for amplicon sequencing

| **PCR amplification of *GBSSI* alleles** | | | | **Region amplified in NC_008399** |
| --- | --- | --- | --- | --- |
| **Primer combinations** | | | **Primer sequences** |  |
| Amplicon 1 | | 1F | CTAGCAGCACAGAGGCACAG | 1.763.647 – 1.764.727 |
|  |  | 15R | CACAAGCAGAGAAGTGAAGCA |  |
| Amplicon 2 | | 7F | GGACCGGGTAAAATGTGTTG | 1.764.519 – 1.765.574 |
|  |  | 18R | ATAAATATCCTGGCCGTTGC |  |
| Amplicon 3 | | 9F | CCGAGTTGGTCAAAGGAAAA | 1.765.273 – 1.766.784 |
|  |  | 31R | GATCTTCTCACCGGTCTTTCC |  |
| Amplicon 4 | | 25F | ACCAGTACAAGGACGCTTGG | 1.766.415 – 1.767.629 |
|  |  | 46R | ATGAGCTCCTCGGCGTAGTA |  |
| Amplicon 5 | | 44F | GAGGGCAGGAAGATCAACTG | 1.767.540 – 1.768.480 |
|  |  | 51R | CCCTGCAGCTGGATGAGT |  |
| Amplicon 6 | | 44F | GAGGGCAGGAAGATCAACTG | 1.767.540 – 1.769.501 |
|  |  | 84R | ATTGGCTATAGCTACAAATT |  |
| **Sequencing** | | | |  |
| **Amplicons** | **Primers** | | **Primer sequences** | |
| Amplicon 1 | 1F | | CTAGCAGCACAGAGGCACAG |  |
|  | 2R | | TGCTTGTACGTGCTGACAAA |  |
|  | 3R | | CGTACGTCGATCGTGTGG |  |
|  | 4F | | CAAACTGGCAGGCACTCAG |  |
|  | 5R | | CTTGGCGTACGTTGCATTG |  |
|  | 6F | | CAAGCTGGAAAAGCAAAAGG |  |
|  | 7F | | GGACCGGGTAAAATGTGTTG |  |
|  | 13R | | GTTTCCCCCGTTTGTTTTCT |  |
|  | 14R | | CCTCCCGCAACACATTTTAC |  |
|  | 15R | | CACAAGCAGAGAAGTGAAGCA |  |
| Amplicon 2 | 7F | | GGACCGGGTAAAATGTGTTG |  |
|  | 9F | | CCGAGTTGGTCAAAGGAAAA |  |
|  | 16R | | CCCCTGGGTGTGTTTCTCTA |  |
|  | 17R | | TTTCTCCAGTGGCGAGAGAC |  |
|  | 18R | | ATAAATATCCTGGCCGTTGC |  |
| Amplicon 3 | 9F | | CCGAGTTGGTCAAAGGAAAA |  |
|  | 10F | | TAGCCACCCAAGAAACTGCT |  |
|  | 11F | | TGCAGAGATCTTCCACAGCA |  |
|  | 18R | | ATAAATATCCTGGCCGTTGC |  |
|  | 19R | | CAATTTGGATTGGGGATTAGAA |  |
|  | 20F | | GCTAGACAACCACCATGTCG |  |
|  | 21R | | GCTGGTCGTCACGCTGAG |  |
|  | 22F | | GCATGAACGTCGTGTTCG |  |
|  | 23R | | GCGATCGAAGTTTGTGTGTG |  |
|  | 24F | | TTCTTGATCATCGCATTGGA |  |
|  | 25F | | ACCAGTACAAGGACGCTTGG |  |
|  | 26R | | GCTCCTACCTCAGCCACAAC |  |
|  | 27F | | TTCGCAAGATTTTAACCCAAG |  |
|  | 28R | | CTCCACCTTCTCCAGGAATG |  |
|  | 29F | | GCAGATCAAGGTTGCAGACA |  |
|  | 31R | | GATCTTCTCACCGGTCTTTCC |  |
| Amplicon 4 | 25F | | ACCAGTACAAGGACGCTTGG |  |
|  | 28R | | CTCCACCTTCTCCAGGAATG |  |
|  | 29F | | GCAGATCAAGGTTGCAGACA |  |
|  | 30F | | GTTCATCGACCATCCGTCAT |  |
|  | 32F | | TGATTTCAGGTTTGGGGAAA |  |
|  | 33R | | TGGTAAACTCGTGATGCTTCC |  |
|  | 35R | | TCAATTGTAACTCACCATAAGTTCCT |  |
|  | 36F | | TACCTGCAGGCAGCACTC |  |
|  | 38F | | AGGTGAGGATGTTGTGTTCG |  |
|  | 39R | | CAGTGTGCATGCAGATTTGA |  |
|  | 40F | | TCACTGCAGGTTGCTTTCTG |  |
|  | 42R | | TGAAATCGAAGGATGACCTG |  |
|  | 43F | | CAACCCTGCACTACTGTCCA |  |
|  | 46R | | ATGAGCTCCTCGGCGTAGTA |  |
| Amplicon 5 | 44F | | GAGGGCAGGAAGATCAACTG |  |
|  | 45R | | TCGTACTTGGCGGTGATGTA |  |
|  | 48R | | TTCGTTCTTACCGTGGTTGC |  |
|  | 49R | | CTTCCGGCTAACTCCACAAG |  |
|  | 50F | | TCCGTGTGTGTTTCAGGGTA |  |
|  | 51R | | CCCTGCAGCTGGATGAGT |  |
| Amplicon 6 | 44F | | GAGGGCAGGAAGATCAACTG |  |
|  | 45R | | TCGTACTTGGCGGTGATGTA |  |
|  | 48R | | TTCGTTCTTACCGTGGTTGC |  |
|  | 49R | | CTTCCGGCTAACTCCACAAG |  |
|  | 50F | | TCCGTGTGTGTTTCAGGGTA |  |
|  | 51R | | CCCTGCAGCTGGATGAGT |  |
|  | 52R | | GCATTGCATTGCACAAAGAT |  |
|  | 53F | | CAAATGCAAATGCATGATGA |  |
|  | 55R | | CCGACTTATCGGTATTAGGATG |  |
|  | 56F | | TTTTTGGCAGTGCAAGGTG |  |
|  | 57R | | TTCGTAATTTATACCTTCCAGGAGA |  |
|  | 58R | | GAATATTTAGAAGCGGAGGGAGT |  |
|  | 60F | | TTTGCAACATGGATTTCAGG |  |
|  | 61R | | AGATCTCAGGCTCTTCAAGGA |  |
|  | 70F | | ACAAGACGAACGGTCAAACA |  |
|  | 71F | | TTCTTTTCCTATCATTTGATTCATTG |  |
|  | 84R | | ATTGGCTATAGCTACAAATT |  |
